# Largest Neck Circumference Associated With Obstructive Sleep Apnea: A Case Report

**DOI:** 10.7759/cureus.54761

**Published:** 2024-02-23

**Authors:** Mohamed Bilal Haradwala, Manjamalai Sivaraman

**Affiliations:** 1 Department of Neurology, University of Missouri, Columbia, USA

**Keywords:** obesity-related illnesses (oris), risk factors for obstructive sleep apnea (osa), obesity hypoventilation syndrome, circumference of the neck, obstructive sleep apnea (osa)

## Abstract

Obstructive sleep apnea (OSA) is a sleep-related breathing disorder associated with obesity markers such as neck circumference (NC), waist circumference (WC), and body mass index (BMI). A greater NC has been linked to a higher risk of OSA. To date, the largest reported NC has been 20.5 inches.

We present a case of a patient who came to our clinic for OSA management and was found to have an NC of 25 inches, the largest reported to date.

The primary objective of this case is to stimulate discussion and research on the specific limits of NC that indicate a higher risk of OSA, the impact of extreme obesity on the severity of the condition and its treatment challenges, and the importance of basic physical measurements of NC in assessing a patient's level of risk and providing appropriate care.

This case highlights an extreme clinical observation of a common medical problem. It illustrates the importance of physical examination findings and their relevance in OSA.

## Introduction

Obstructive sleep apnea (OSA) is a sleep-related breathing disorder characterized by the recurrent collapse of the pharyngeal airway, resulting in obstructive events that necessitate arousals to reestablish airway patency [1]. The association between OSA and various anthropometric measurements, particularly obesity-related parameters, has been the subject of extensive research, highlighting the importance of identifying individuals at risk. Objective measures of obesity, such as neck circumference (NC), waist circumference (WC), and body mass index (BMI), have been utilized to identify the risk of OSA. Patients with OSA have been observed to have a higher NC [2].

We present a case where a patient had been diagnosed with OSA and was found to have an NC of 25 inches. Based on our search of MEDLINE using PubMed with the search terms "obstructive sleep apnea" and "neck circumference," we did not find any prior reports of an NC of this magnitude.

## Case presentation

A 51-year-old male patient was referred to the sleep disorders center for the management of OSA due to his inability to tolerate continuous positive airway pressure (CPAP). The patient had a medical history that included type 2 diabetes mellitus, asthma, hypertension, and obesity with a BMI of 43.6. He presented with snoring, witnessed apneas, and excessive daytime sleepiness. His family history did not include any known cases of sleep apnea, and he denied using tobacco or alcohol.

He had been diagnosed with sleep apnea in the year 2020 based on in-lab diagnostic polysomnography, which revealed severe OSA with an apnea-hypopnea index (AHI) of 41.2, as per the 4% desaturation criteria. He experienced a minimum oxygen saturation of 52%, with 111.2 minutes spent below an oxygen saturation of 89%. Subsequent trials of bilevel-positive airway pressure (BiPAP) therapy at home were unsuccessful due to intolerance.

Given his inability to tolerate positive airway pressure (PAP), the patient expressed interest in using hypoglossal nerve stimulator therapy as a treatment option for his OSA.

**Figure 1:**
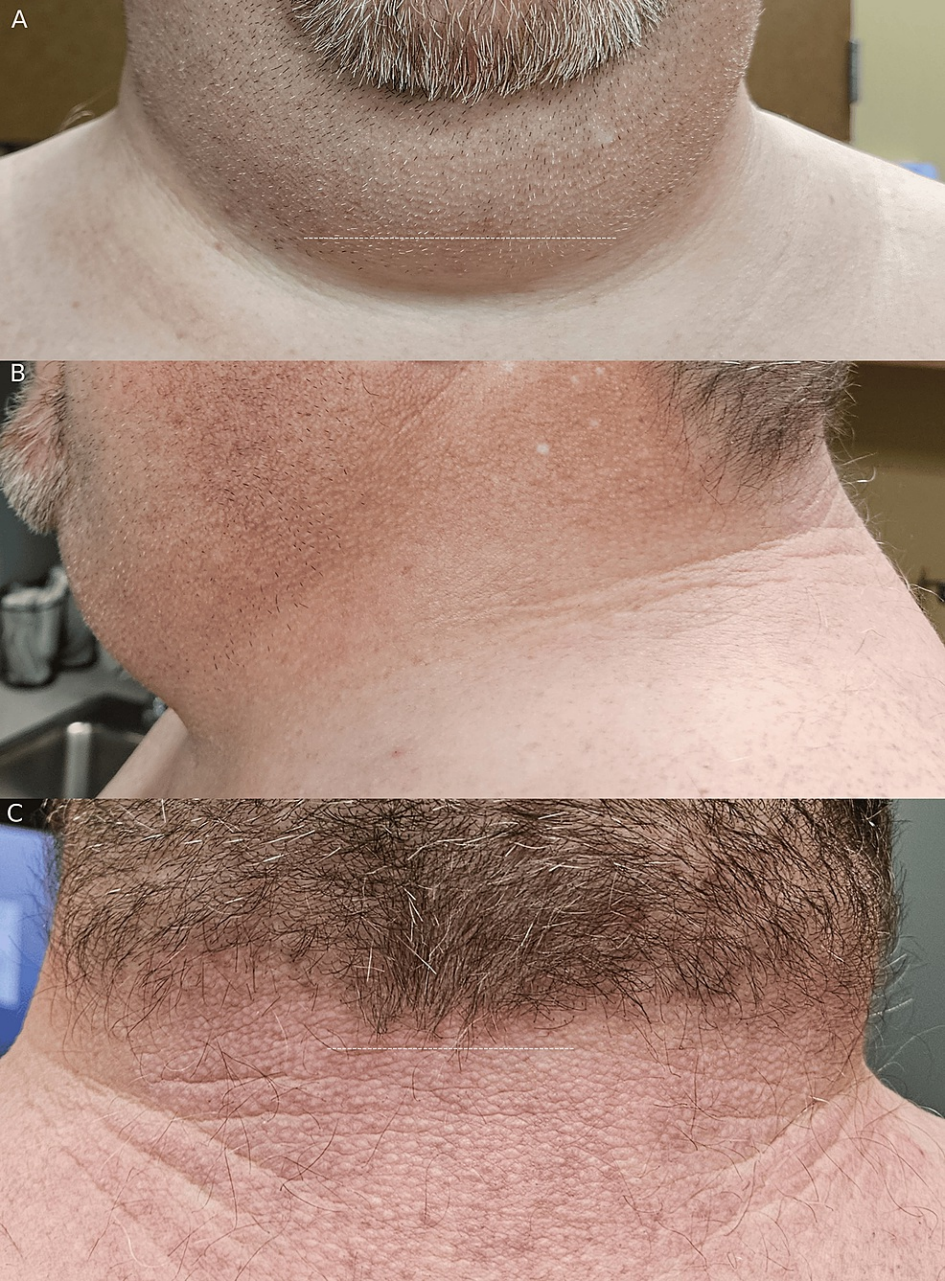
(A) Anterior view of the patient's neck where a white dotted line depicts the position of the superior border of the tape measure approximately below the level of the laryngeal protuberance assessed by palpation. (B) Left lateral view of the patient's neck without tape. (C) Posterior view of the patient's neck without tape where a white dotted line depicts the position of the superior border of the tape measure.

On physical examination, the patient was awake, alert, and oriented. He exhibited no difficulties in speech or comprehension. As part of a comprehensive sleep evaluation, his upper airway was examined, including his NC, which was measured using a commercial measuring tape due to standard measuring tapes from Medline being insufficient. The superior border of the tape measure was placed just below the laryngeal prominence, which was difficult to palpate due to the increased adiposity of his neck, as illustrated in Figure 1. During the measurement, he was standing erect with his head positioned in the Frankfort horizontal plane as described in the Framingham Heart Study [3].

**Figure 2:**
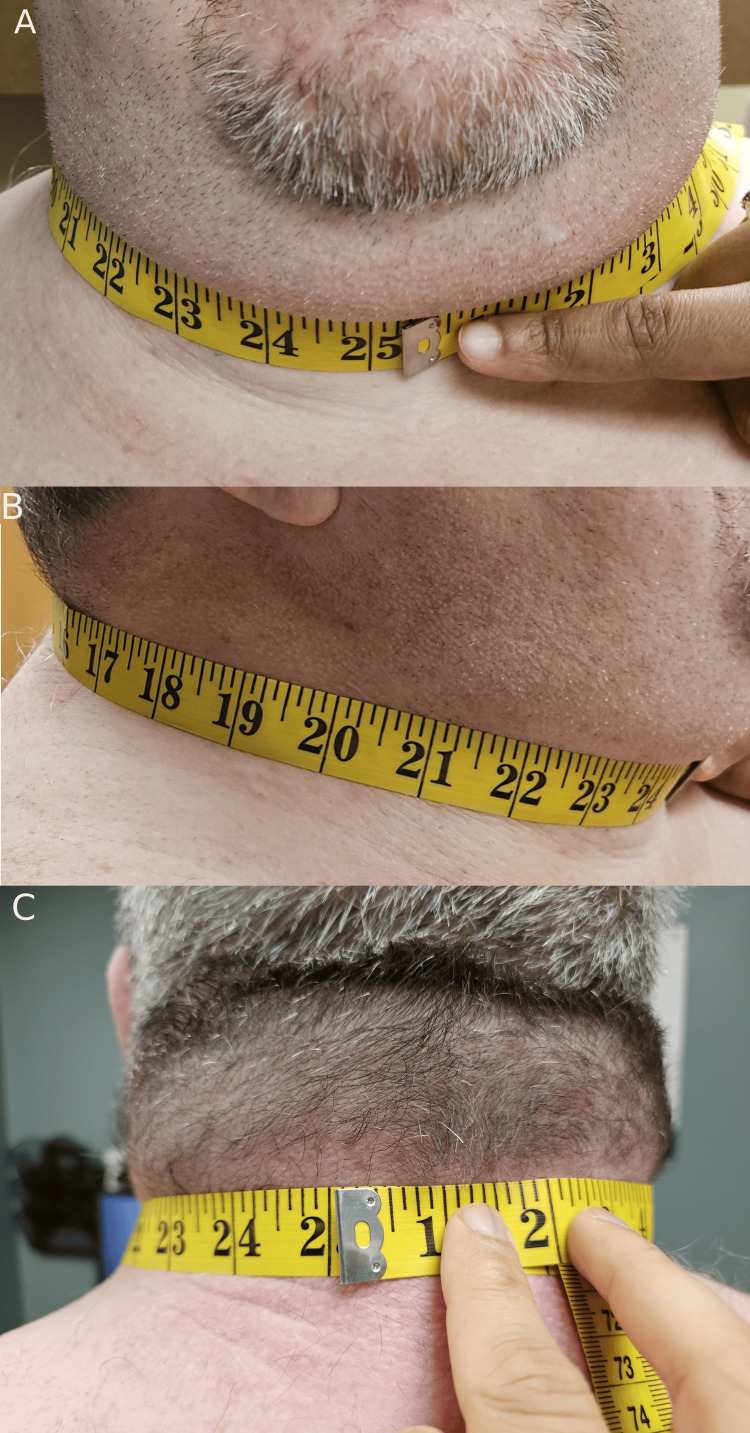
(A) Anterior view of measuring tape just below the laryngeal protuberance measuring 25 inches (rounded to the nearest quarter inch). (B) Right lateral view of patient's neck with measuring tape. (C) Posterior view of the patient's neck with measuring tape positioned perpendicular to the long axis of the neck.

Our patient's NC measured 25 inches (rounded to the nearest quarter inch), as illustrated in Figure 2. Images of our patient were captured after obtaining consent.

## Discussion

OSA has been associated with obesity due to its influence on the upper airway [1]. Commercial motor vehicle drivers with a BMI greater than 33 kg/m2 are at significantly elevated risk for OSA even in the absence of reported symptoms [4]. Neck size has been reported as a "vital sign," and patients with a neck size of 17 inches (43 cm) or more in men and 16 inches (41 cm) or more in women have an increased risk of developing OSA [5]. A study conducted in Cincinnati Veterans Affairs Medical Center on 596 patients revealed increasing severity of OSA with increasing NC, BMI, Epworth Sleepiness Scale (ESS) scores, hypertension, congestive heart failure, and type 2 diabetes [6]. Another study at the Federal University of Minas Gerais showed an increasing odds ratio of NC associated with OSA severity, i.e., 1.1592, 1.2451, and 1.5706 with mild, moderate, and severe OSA, respectively [7].

Our case of a 51-year-old male patient with a history of OSA and a measured NC of 25 inches (Figure 2) offers an opportunity for discussion regarding several important aspects of OSA, its clinical assessment, and its management. Anthropometric data obtained in studies have shown that NC is associated with an increased risk of developing metabolic syndrome as well as OSA [8,9]. To date, no prior case of an NC of 25 inches has been recorded in association with OSA. According to the records, the neck with the greatest thickness measures 20.5 inches [10]. We also searched PubMed using MEDLINE and did not identify any prior reports of such a large neck size.

## Conclusions

Based on our search of MEDLINE using PubMed, this is the first case report of a patient with such a large NC that has been associated with OSA. Our patient's vast NC, as measured at 25 inches (Figure 2), is a striking illustration of the potential of NC as a predictive marker for OSA. This should be measured in all OSA patients during the initial evaluation.
